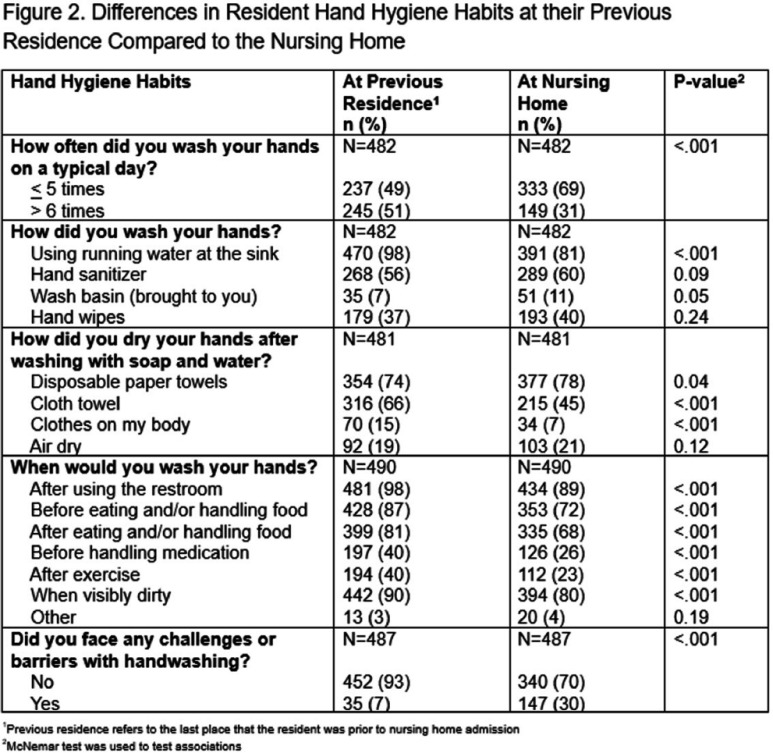# Survey of Nursing Home Resident Hand Hygiene Knowledge and Behavior

**DOI:** 10.1017/ash.2025.334

**Published:** 2025-09-24

**Authors:** Hailey Blinkiewicz, Yasin Abul, Jessica Ameling, Christopher Crnich, Jazmin Duque, JJ Furuno, Stefan Gravenstein, Morgan Katz, Jennifer Meddings, Sujan Reddy, Nicole Sandberg, Emily Short, Chin-Lin Su, Lona Mody, Ganga Vijayasiri

**Affiliations:** 1 University of Michigan; 2 Brown Alpert Medical School Division of Geriatrics; 3 University of Wisconsin; 4 Abt Global; 5 Oregon State University College of Pharmacy; 6 Brown University and Providence Veterans Administration Medical Center; 7 Johns Hopkins University; 8 CDC; 9 Oregon State University College of Pharmacy

## Abstract

**Background:** Hand hygiene is an important strategy for reducing healthcare-associated infections. While efforts to improve nursing home (NH) staff hand hygiene have been prioritized, there are few if any policies in-place to improve resident hand hygiene. Further, CMS guidance requires that residents be bathed “twice a week.” The objective of this study was to characterize resident hand hygiene knowledge and habits as well as bathing practices to identify barriers in a setting where new intervention strategies could be aimed. **Methods:** The survey was administered at 20 NHs across the United States between December 2023 and July 2024. Verbal consent was obtained from residents before survey administration. Survey questions explored residents’ hand hygiene knowledge and differences in hand hygiene habits and bathing practices since entering the NH from their last place of residence. Three knowledge-based questions assessed residents’ understanding of the recommended length of time to wash hands and use hand sanitizer in addition to when hand washing should be utilized instead of hand sanitizer. Frequency of hand washing, either through soap and water or hand sanitizer, instances of when and how residents wash and dry their hands, and whether resident’s faced challenges were assessed. **Results:**Of the 495 residents who completed the survey, only 142 (29%) residents answered all three knowledge-based questions correctly. Residents who answered two or three questions correctly reported washing their hands more frequently at their previous residence compared to residents who answered zero or one correct (Figure 1). Frequency of hand hygiene was lower at the NH compared to their previous residence across a variety of indications (Figure 2). More residents faced challenges with washing their hands at the NH compared to their previous residence (30% vs. 7%, P<.001). The most common challenges included: mobility limitations, medical issues, need for assistance, bathroom accessibility and inadequate bathroom supplies/equipment. About half the residents (53%) reported never being reminded to wash their hands; 60% reported that they would use hand sanitizer if it was easily accessible. 51% of residents reported bathing with soap and water less at the NH compared to their previous residence with reported causes being needing help and not receiving it, nursing home policy, medical issues, and mobility limitations. **Conclusions:** Survey results indicate opportunities for interventions aimed at reducing the barriers to hand hygiene practice and improving bathing practices in NHs. Policy changes and hand hygiene educational opportunities addressing these barriers could serve as potential strategies.

**Figure:**
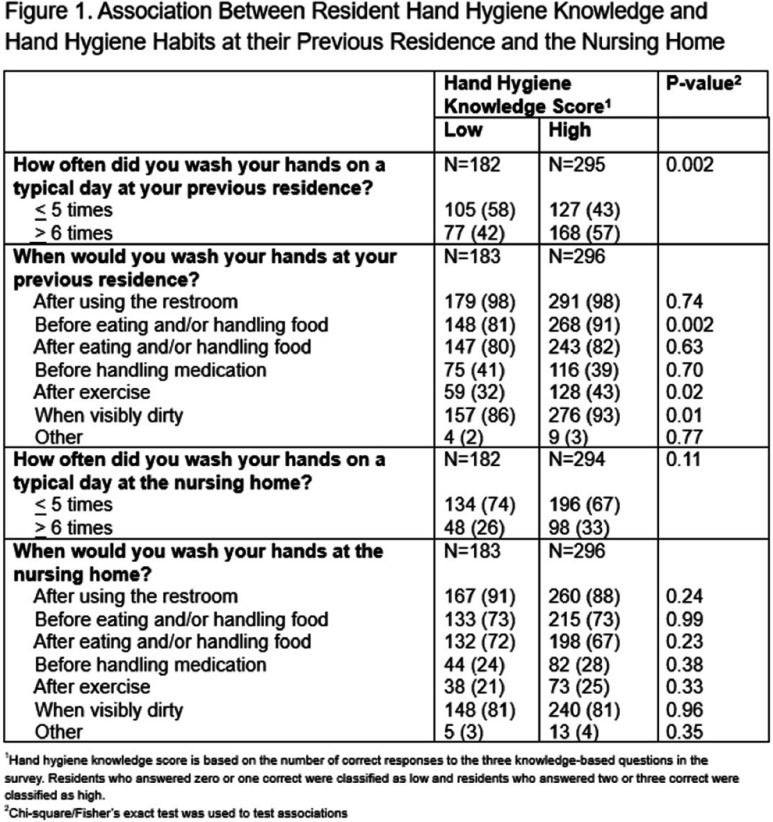

**Figure:**